# Analogy between Acute Idiopathic Pleuritis and Acute Articular Rheumatism

**Published:** 1887-09

**Authors:** 


### *ANALOGY BETWEEN ACUTE IDIOPATHIC PLEURITIS AND ACUTE ARTICULAR RHEUMATISM.*

Dr. E. L. Shurly, of Detroit, read an instructive paper on this subject before the recent meeting of the Michigan State Medical Society. He shows that the ordinary etiology, viz., exposure to cold, is unsatisfactory in explaining the development of acute pleuritis, and relates the instance of three apparently equally healthy men remaining all night in the forest in the autumn with the following results: One was attacked with acute pleuritis, one with acute rheumo-arthritis, while the third escaped disease.

The same cause—exposure to cold—is set down as an important factor in the development of acute articular rheumatism, but the vital change taking place between the "exposure" and the resulting disease is yet shrouded in mystery. The author then goes on to show that we must look for some antecedent constitutional condition in order to explain the different results arising from the same apparent cause. He finds this antecedent condition to be the rheumatic diathesis, the working of the morbific agent being now turned towards the pleuræ, now towards the serous membranes of the joints. He arrives at this conclusion in three ways:

1. Histologically, all the fibro-serous membranes are identical, all proceeding from the meso-blast, or middle of the primordial layers, and ultimately developing into the endothelial layer, or lining proper of all closed cavities. This is situated upon a basement membrane, proliferated, probably, by the fibrous tissue or layer surrounding it, and constituting the framework. To this endothelial layer in all cases belongs the secretory property of the part, which we know varies, as, for example, the presence of mucin in the synova. The statement of some histologists, that in the case of the joints, the fibrous, basement and synovial layers are proliferated from the outlying cartilage, does not alter the case, because the histological characters of the fully-formed tissues are the same; neither does the mode of origin and arrangement of the lymphatics, which seems to be an undecided question, for we know that each fibro-serous membrane must have, and does have, its lymphatic system in one form or another. Therefore, we must accept as a fact that these several serous membranes belong to the same anatomical system.

2. Pathologically, also, the identity of these diseases is well marked. In acute pleuritis, pericarditis, peritonitis and rheumatic or simple synovitis, the steps of the disease process are the same, or nearly so, viz.: hyperæmia, effusion (either fibro-serous, lymph, blood, pus, or a mixture of these). The ultimate disposal of the effused material is nearly the same for each locality; for there may be complete or partial absorption, calcareous or fatty degeneration, adhesion and pyogenesis in either disease.

3. Clinically, the history of these diseases exhibit close resemblances—the degrees of constitutional or local disturbance, the mode of attack, the intermittent or remittent or continued character of their course, or pyrexia and their complications. Lastly, the evidence from treatment is important, many cases of acute idiopathic pleuritis yielding only after a course of anti-rheumatic medication.

This imperfect review does not do justice to the paper, which is worth reading in its entirety.

